# The Effects of a 10-Week Water Aerobic Exercise on the Resting Blood Pressure in Patients with Essential Hypertension

**DOI:** 10.5812/asjsm.34854

**Published:** 2010-09

**Authors:** Ali Vasheghani Farahani, Mohammad-Ali Mansournia, Hossein Asheri, Akbar Fotouhi, Masud Yunesian, Mohsen Jamali, Vahid Ziaee

**Affiliations:** 1 Sports Medicine Research Center, Tehran University of Medical Sciences, Tehran, IR Iran; 2 Department of Cardiology, Tehran University of Medical Sciences, Tehran, IR Iran; 3 School of Public Health, Tehran University of Medical Sciences, Tehran, IR Iran

**Keywords:** Water aerobic exercise, Swimming, Essential hypertension, Blood pressure

## Abstract

**Purpose:**

To investigate the effects of a 10-week water aerobic exercise on the resting blood pressure in patients with stage 1 or 2 hypertension referring to Tehran University Clinics.

**Methods:**

Forty men with stage 1 or 2 essential hypertension were assigned to two groups of intervention [n = 12; aged 48.33±10.74 years (mean±SD)] and control [n = 28; aged 46.96±11.58 years (mean±SD)]. Subjects in the intervention group participated in a supervised 10-week water aerobic training program of 55 min sessions, 3 days per week on alternate days, while those in the control group were not involved in any regular training program during this period. Blood pressure of the participants was recorded and compared at the beginning and at the end of the study (48 hours after the last training session).

**Results:**

Exercise lowered systolic blood pressure and mean arterial pressure by 11.71 (95% confidence interval: 5.07 to 18.35) and 5.90 (95% confidence interval: 1.17 to 10.63) mm Hg respectively. The lowering effect of exercise on diastolic blood pressure was neither statistically significant nor clinically important (0.55 mm Hg; P. value = 0.8). There was no significant effect of age, baseline body mass index and stage of hypertension on the exercise-induced changes in blood pressure.

**Conclusion:**

A 10-week course of water aerobic exercise markedly reduced the systolic and mean arterial blood pressure of patients with essential hypertension and is especially recommended for the obese and the elderly who have orthopedic problems or bronchospasm.

## INTRODUCTION

Hypertension, defined as resting systolic blood pressure of >= 140 and/or diastolic blood pressure of >= 90 mmHg, remains the most common risk factor for cardiovascular morbidity and mortality. Currently, more than a quarter of the world adult population suffer from hypertension and it is expected that this number will increase to 1.56 billion (29%) by 2025^[1, 2]^. The primary goal of the treatment of hypertension is to prevent cardiovascular disease and mortality^[3]^. All guideline statements emphasize life style modification as the first step in the treatment of hypertension. In this regard, physical activity is recommended for the hypertensive patients as a part of life style modification^[4, 5]^.

Several clinical trials have studied the effects of dynamic aerobic exercises on resting blood pressure. The meta-analyses of these studies support the role of aerobic exercises in prevention and treatment of hypertension^[6–8]^. The majority of these studies have used walking, jogging, cycling, running or a combination of these activities as training modalities. In addition to these sports, swimming has been widely recommended by authoritative guidelines as an appropriate means of the prevention and treatment of hypertension^[9–12]^. Nevertheless, evidence arising from scientific investigation on the effect of swimming on blood pressure is scant. Swimming is an inexpensive, available and attractive sport with a dynamic nature. The minimal weight-bearing stress, humid environment, and a reduced heat load makes swimming especially convenient for overweight individuals and those with exercise induced bronchospasm and/or orthopedic problems^[13, 14]^.

Some cross-sectional studies suggest that swimming training might not be effective in modifying the resting blood pressure^[15–18]^. According to these studies, blood pressure and heart rate increase during immersion and swimming, particularly in the elderly^[15]^. Also for the same heart rate, mean arterial blood pressure is higher during swimming compared to running^[16]^. Moreover, systolic and diastolic blood pressure in trained swimmers is higher than in other endurance-trained athletes^[17, 18]^.

Distinguishing features of swimming such as supine body position, facial immersion, hydrostatic pressure exerted on the athlete, possible effects of water temperature and increased thermal conductivity of water can merely explain the immediate increase in blood pressure following swimming^[13, 19]^. In any case, longitudinal trials investigating the long-term (and not acute) effect of swimming on blood pressure are few and somewhat controversial^[14, 19]^. Considering the paucity of evidence in this area, this study aimed to investigate the effects of water aerobic training on blood pressure in hypertensive patients with stage 1 or 2 hypertension referring to Tehran University Clinics.

## METHODS AND SUBJECTS

Among patients referred to Tehran University Clinics in Tehran in 2006, forty individuals who had received the diagnosis of mild or moderate hypertension (140≤ systolic blood pressure <180 or 90≤ diastolic blood pressure <110) by a cardiologist and met the inclusion criteria entered the study. Inclusion criteria consisted of between 20 and 70 years of age, diagnosis of essential hypertension after clinical and laboratory exclusion of secondary hypertension by a cardiologist, absence of severe or refractory hypertension, absence of evidence of target organ damage (i.e. heart, eye, kidney, and brain), no evidence of coronary artery disease (normal clinical history, physical examination, electrocardiogram and exercise stress test) and absence of any chronic illness that may limit the patient's capacity to participate in exercise. The patients were excluded from the study if they were absent for more than two sessions from the training or if they started drug therapy or developed complications like unstable angina, myocardial infarction, or cerebrovascular accidents.

The participants were assigned to two groups of intervention and control. Both groups were recommended to reduce weight, to limit salt consumption, and to stop smoking and drinking alcohol. Subjects in the intervention group participated in a supervised 10-week water aerobic training program of 55 min sessions, 3 days per week on alternate days in addition to the general recommendations. Blood pressures were measured using mercury sphygmomanometers (Richter, Germany) in the sitting position according to the standard guidelines for measurement of blood pressure by sphygmomanometry. Briefly after the subjects rested for five minutes in the seated position, a minimum of 2 readings were taken at intervals of at least 1 minute and the average of those readings was used to represent the patient's blood pressure. If there was >5 mmHg difference between the first and second readings, additional (1 or 2) readings were obtained, and then the average of these multiple readings was used. Blood pressure was measured at baseline and 48 hours after the last exercise session to avoid the acute effects of a single bout of exercise. Each training session consisted of warm-up (10 min), water aerobic training (25 min), swimming skills training (10 min), and cool-down (10 min).

Participants’ heart rates were recorded after the warm up and each training phase. The exercise intensity was set at 60-65% of the maximal heart rate and increased gradually up to 70-75% during the program. Maximal heart rate was adjusted on the basis of the observation that the maximal heart rate during swimming is 10–13 beats lower compared with land-based activities. The heart rate of individuals participating in aerobic exercise is expected to rise from 60-65% to 70-75% of maximum capacity. It is notable that knowing how to swim was not necessary to enter the study but the participants acquired the skills during the ten weeks of training. The study was approved by the ethics committee of Tehran University of Medical Sciences and written informed consent was obtained from each patient.

Continuous variables [blood pressure, heart rate, and body mass index (BMI)] were summarized for each group by the mean and standard deviation [presented as mean (SD)]. Baseline values of systolic and diastolic blood pressure are potentially confounding factors in analyzing the effects of exercise. In order to improve the precision of the estimated effects and to achieve an unbiased estimate and confidence interval for the magnitude of effect, we used Analysis of Covariance (ANCOVA), assuming baseline systolic and diastolic blood pressure values as the covariates^[21, 22]^. The assumptions of ANCOVA including lack of interaction between variables, i.e. baseline values of blood pressure and treatment group, normal distribution of studentized residuals (based on normal Q-Q plot) and constancy of residual variance (based on the residuals plot against the fitted values and also against each of the explanatory variables such as baseline blood pressure values) were assessed. For inferential purpose 95% Confidence intervals were presented for the contrast between groups (the variable coefficient in covariate analysis model). We also used Bayesian statistics to combine existing evidence of the effects of water aerobic exercise on blood pressure with the present data^[23, 24]^.

The sample size was estimated based on change score analysis. According to the study by Tanaka et al, and considering a type I error probability of 5%, the power of this study in detecting a 5 mmHg reduction in systolic blood pressure is 80%^[14]^.

## RESULTS

The baseline characteristics of the patients in the intervention and control groups are depicted in Table 1. Systolic blood pressure, diastolic blood pressure and mean arterial blood pressure at the end of study (48 hours after the last exercise session) were 137.5 (9.65), 92.08 (9.15) and 107.22 (8.86) mmHg respectively. In the control group, systolic blood pressure, diastolic blood pressure and mean arterial blood pressure at the end of study were 144.82 (10.49), 91.07 (5.15) and 108.98 (5.86) mmHg respectively.

**Table 1:** Baseline characteristics of the patients in the intervention and control groups

Variable	Intervention group (n = 12)	Control group (n = 28)
**Age (year)**	48.33 (10.74)*	46.96 (11.58)
Systolic BP**(mmHg)	154.17 (10.84)	146.6 (8.50)
**Diastolic BP (mmHg)**	96.25 (5.69)	90.89 (5.78)
**Mean arterial BP (mmHg)**	115.55 (5.92)	109.46 (4.97)
**BMI (kg/m^2^)**	27.44 (4.27)	28.06 (3.51)
**Heart rate (beat/min)**	67.17 (6.23)	73.00 (7.05)

* Mean (SD)

** BP: Blood Pressure

**Fig. 1:**
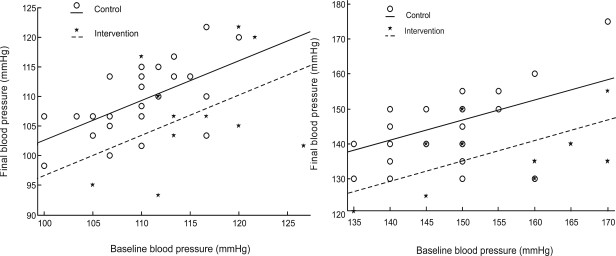
ANCOVA Plot for systolic blood pressure (right) and mean arterial blood pressure (left).The X and Y axes represent subjects' blood pressure at baseline and at the end of the study respectively

In ANCOVA, the coefficient of group variable (taking the value 1 for intervention and 0 for control group) is the effect of interest: the estimated difference between the two treatment groups (Table 2). As a type of multiple regression models, ANCOVA provides a means of prediction (Fig. 1). For systolic blood pressure, the regression equation is:

Z=59.73−11.7X+0.58Y

**Table 2:** The estimates of blood pressure reduction with 95% confidence interval

Variable	The coefficient of group variable	P value	95% Confidence Interval
**Systolic BP (mmHg)**	−11.71	<0.001	−18.35 to −5.07
**Diastolic BP (mmHg)**	−0.55	0.8	−5.46 to 4.36
**Mean arterial BP (mmHg)**	−5.90	0.016	−10.63 to −1.17

BP: Blood Pressure

where X, Y, and Z represent group, baseline systolic blood pressure, and final systolic blood pressure values respectively. Based on this equation, a patient who has a systolic blood pressure of 140 mmHg at the time of entering the 10-week course of training will have a final systolic blood pressure of 129 mmHg. In Fig. 1, the magnitude of blood pressure reduction, estimated by ANCOVA, equals the vertical distance between the two parallel lines.

It is notable that the results shown in Table 2 did not change significantly after including age and BMI as covariates in regression model. Four patients in the control group were being administered anti-hypertensive medication (beta blocker drugs). The results were not affected by the inclusion or exclusion of these subjects. Table 3 summarizes the estimates of blood pressure reduction and 95% confidence interval based on age, BMI and stage of hypertension.

**Table 3:** Estimates of blood pressure reduction based on age, BMI and stage of hypertension

Variable	Systolic BP (mmHg)	Diastolic BP (mmHg)	Mean arterial BP (mmHg)
**Age < 50 (year)**	−12.67(−21.45to −3.8)*	0.04 (−5.82 to 5.91)	−8.06 (−14.47 to −1.66)
**Age > 40 (year)**	−9.62 (−22.48 to 3.23)	1.74 (−6.10 to 9.59)	−2.79 (−11.15 to 5.57)
**BMI < 25 (kg/m^2^)**	−8.17 (−19.07 to 2.73)	3.75 (−9.49 to16.99)	−2.75 (−12.71 to 7.20)
**BMI > 25 (kg/m^2^)**	−11.83( −19.8 to 3.78)	−2.86 (−8.78 to 3.05)	−7.56 (−13.49 to–1.63)
**Stage I hypertension**	−6.26 (−14.91 to 2.38)	0.31 (−5.32 to 5.94)	−2.65 (−7.68 to 2.39)
**Stage II hypertension**	−16.9 (−31.50to−2.42)	−2.38(−14.23to 9.47)	−8.33 (−19.49 to −2.83)

* 95% confidence interval/BMI: Body Mass Index/BP: Blood Pressure

According to data analysis, the minimum Bayes factors were 0.005, 0.97, and 0.055 for systolic blood pressure, diastolic blood pressure, and mean arterial blood pressure respectively (Table 4).

**Table 4:** Bayesian interpretation of *P* value and its effect on the probability of null hypothesis

Variable	Prior probability	Minimum Bayes factor	Posterior probability	Strength of evidence
**Systolic BP (mmHg)**	0.50	0.005	0.005	Strong to very strong
**Diastolic BP (mmHg)**	0.50	0.97	0.49	zero
**Mean arterial BP (mmHg)**	0.50	0.055	0.05	Moderate to strong

BP: Blood Pressure

## DISCUSSION

Swimming has been specifically and widely recommended for the prevention and treatment of hypertension by the relevant national and international health organizations^[9–12]^. Early recommendations (before 1997) have been extrapolated from the effects of other aerobic exercise forms (such as walking, jogging, and cycling) and are not based on scientific evidence^[12, 25–27]^. Current recommendations (after 1997) are based primarily on the results of a trial involving 18 men and women with hypertension, of whom 12 were assigned to a 10-week swimming training program done by Tanaka et al^[9–11]^. Most people enjoy swimming and water aerobic exercises due to its rhythmic and dynamic nature and also because of its humid and minimal weight-bearing stress environment. These activities are particularly useful for people who have orthopedic problems or bronchospasms, or those who are obese^[13, 14]^. The present study is among the first to examine the effects of water aerobic exercise on the resting blood pressure in individuals with essential hypertension.

Our data suggest that a 10-week course of water aerobic exercises (including swimming training) reduces the systolic and mean arterial blood pressure of the participants by 11.71 and 5.9 mmHg respectively. Based on this study, a 10-week water aerobic exercise has an impressive effect on the resting systolic blood pressure in individuals with hypertension. 5 mmHg reduction of systolic blood pressure, the lower bound of confidence interval in the population would result in a 14 percent overall reduction in mortality due to stroke, a 9 percent reduction in mortality due to coronary heart disease, and a 7 percent decrease in all-cause mortality^[3]^. Considering the confidence interval, the effect of exercise on mean arterial pressure reduction is also significant. Exercise as a lifestyle modification can be recommended for all patients with hypertension, since even small reductions in blood pressure are associated, in long-term, with a reduced risk of cardiovascular diseases^[28]^.

Although we could not reject the null hypothesis that "water aerobic exercise has no effect on resting diastolic blood pressure", it must be considered that: 1) Our sample size was calculated based on an estimated systolic blood pressure reduction. We do not have adequate power to detect real and clinically worthwhile diastolic blood pressure reduction. Confidence interval suggests that the result is inconclusive. 2) Diastolic blood pressure is a more potent cardiovascular risk factor than systolic blood pressure until the age of 50 and systolic blood pressure is more important thereafter. The greatest burden of hypertension-related cardiovascular disease at present occurs in the middle-aged and elderly, in whom systolic hypertension predominates^[29]^.

Subgroup analysis reveals that there is no significant effect of age, baseline BMI and stage of hypertension on the exercise-induced changes in blood pressure, which is in accordance with the results of meta-analyses by Whelton and colleagues (2002) and Fagard and colleagues (1999) (Table 3)^[6, 8]^. However sparsity of comparison cells makes it difficult to draw conclusions based on the subgroup analysis.For example, only five patients in the control group had stage II hypertension and overall, only eight patients had BMI less than 25.

The minimum Bayes factor for systolic blood pressure (i.e. 0.005) is so powerful that can reduce the 0.92 probability of the null hypothesis, lack of effect of water aerobic exercises on systolic blood pressure, down to 0.05 (Table 4). Considering the mechanisms proposed for the effect of aerobic exercises on blood pressure and the results of trials focused on the influence of aquatic aerobic exercises on blood pressure, we pessimistically assumed a prior probability of 0.50 for the null hypothesis (equivalent to the simple experiment of tossing a fair coin). Our findings reduced this probability to 0.005 percent. The posterior probability of a true null hypothesis for mean arterial blood pressure and diastolic blood pressure were 0.05 and 0.49 respectively. These results consistent with our previous knowledge are strong enough to conclude that water aerobic exercise can efficiently reduce systolic and mean blood pressure.

Our findings are in accordance with the results of the first randomized clinical trial examined the effects of swimming on blood pressure in hypertensive patients (Tanaka et al, 1997). In their trial, training lowered seated systolic blood pressure by 6 mmHg, on average, and resting heart rate by 10 beats/min, while diastolic blood pressure did not change significantly^[14]^. The intensity and duration of the training were similar to those of our study, but the main part of the exercise session was dedicated to swimming workout. On the other hand, in contrast to our study, most subjects were able to swim continuously for at least 10 minutes at recruitment. Moreover, while in the study by Tanaka et al participants were both men and women and the water temperature was kept constant between 27-28^o^C, our study included only men and was performed at a temperature of 31-32^o^C. The less the temperature of water, the more will the superficial skin vessels constrict and hence the more will the total peripheral resistance increase; this might be a reason for less reduction of blood pressure in Tanaka et al's study.

Cox and colleagues (2006) have performed a randomized controlled trial in which 116 healthy sedentary women, aged 50–70 years, entered randomly into a 6-month program of either swimming or walking exercise of similar intended intensity. Their results indicated that compared to walking, regular swimming significantly elevates blood pressure in previously sedentary, normotensive, older women^[20]^. However, their study differed in several ways from our intervention: Firstly, their study shows the relative effects of swimming on blood pressure compared with walking but they could not determine the absolute effects of swimming on blood pressure that we are looking for. Secondly, the participants in Cox et al's study were all women with different levels of blood pressure, from normal to high. Additionally, some but not all of hypertensive subjects were receiving anti-hypertensive therapy. In the present study, the participants were men with stage 1 or 2 essential hypertension who did not receive anti-hypertensive therapy. It is very likely that aerobic exercise in water has differentially affected these dissimilar target groups. Finally, In Cox et al's report the mean water temperature was 26.5^o^C, whereas the water temperature in our study was 31-32 ^o^C.

Numerous studies have investigated the mechanism of blood pressure reduction after dynamic and aerobic exercise^[30–36]^. Based on these studies decreased total peripheral resistance appears to be the primary mechanism by which resting blood pressure is reduced after exercise training. Reductions in vascular resistance after training are mediated by changes in sympathetic nervous activity, altered vascular responsiveness and changes in vascular structure ^[5, 37–39]^. As an aerobic exercise, swimming could lower the blood pressure through the above mentioned mechanisms, and psychological and stress-reducing effects of aquatic environment may have an additional role. On the other hand, several mechanisms such as supine position assumed during swimming, facial immersion (and stimulation of facial reflex), and a predominance of arm-work have been proposed for elevated blood pressure in swimmers^[13, 20]^. These mechanisms, however, discuss short term and in-event effects; whereas, in general, any aerobic exercise is acutely associated with an increase in cardiac output, a reduction in vascular resistance due to vasodilatation in the exercising muscles, a rise in systolic blood pressure and no change or even a slight reduction in diastolic pressure^[40]^. Most experimental studies, if not all, suggest that swimming lowers both systolic and diastolic blood pressure in hypertensive rats. Increased muscular blood flow followed by a reduction in peripheral vascular resistance, increased insulin sensitivity of muscles, suppression of sympathetic system, and reduction of vasoconstrictor prostaglandins are among the suggested mechanisms^[41–45]^.

The most important limitations of our study are non-random assignment of participants to groups, absence of female participants, and relative paucity of the sample size. These limitations can clearly affect the external and internal validity of the study in an undesirable manner. Values of measurable variables, like baseline values of blood pressure, have been adjusted during analysis but the same could not be done for immeasurable variables (e.g. participant motivation). No evidence exists in recent meta-analyses in favor of a preferential effect of exercise in men^[46]^. As mentioned earlier, the current study enjoys enough power to detect any important reduction (if present) in systolic blood prssure. In general, further randomized trials with adequate sample size are required to more closely investigate the effects of water aerobic exercises on systolic and diastolic blood pressure in hypertensive patients.

## CONCLUSION

Our data suggest that a 10-week water aerobic exercise (supervised by a trainer) has an impressive effect on the resting systolic blood pressure and mean arterial blood pressure in individuals with mild to moderate hypertension. This mode of exercise can be particularly beneficial for elderly individuals with orthopedic problems, overweight individuals and those who have not learned how to swim.
